# Mining Anti-Inflammation Molecules From *Nippostrongylus brasiliensis*-Derived Products Through the Metabolomics Approach

**DOI:** 10.3389/fcimb.2021.781132

**Published:** 2021-11-11

**Authors:** Yuying Chen, Mingming Zhang, Xin Ding, Yougui Yang, Yujia Chen, Qiang Zhang, Yinwen Fan, Yang Dai, Junhong Wang

**Affiliations:** ^1^ Center for Global Health, School of Public Health, Nanjing Medical University, Nanjing, China; ^2^ Key Laboratory of National Health Commission on Parasitic Disease Control and Prevention, Key Laboratory of Jiangsu Province on Parasite and Vector Control Technology, Jiangsu Institute of Parasitic Diseases, Wuxi, China; ^3^ Department of Cardiology, The First Affiliated Hospital of Nanjing Medical University, Nanjing, China; ^4^ Department of Cardiology, The Friendship Hospital of Ili Kazakh Autonomous Prefecture Ili & Jiangsu Joint Institute of Health, Ili, China

**Keywords:** hookworm, *Nippostrongylus brasiliensis*, anti-inflammatory activity, host–pathogen interactions, metabolomics approach

## Abstract

Hookworm is one type of soil-transmitted helminth, which could exert an anti-inflammatory effect in human or animal host, which provides a beneficial possibility for the discovery of inflammatory-related disease interventions. The identification of hookworm-derived anti-inflammatory molecules is urgently needed for future translational research. The emergence of metabolomics has become a powerful approach to comprehensively characterize metabolic alterations in recent times. Herein, excretory and secretory products (ESPs) were collected from cultured adult worm, while small intestinal contents were obtained from *Nippostrongylus brasiliensis* (*N. brasiliensis*, Nb)-infected mice. Through ultra-high-performance liquid chromatography coupled with mass spectrometry (UHPLC-MS) platform, metabolomics analysis was used to explore the identification of anti-inflammatory molecules. Out of 45 differential metabolites that were discovered from ESPs, 10 of them showed potential anti-inflammatory properties, which could be subclassed into amino acids, furanocoumarins, linear diarylheptanoids, gamma butyrolactones, and alpha-keto acids. In terms of intestinal contents that were derived from *N. brasiliensis*-infected mice, 14 out of 301 differential metabolites were discovered to demonstrate anti-inflammatory effects, with possible subclassification into amino acids, benzylisoquinolines, quaternary ammonium salts, pyrimidines, pregnane steroids, purines, biphenyls, and glycerophosphocholines. Furthermore, nine of the differential metabolites appeared both in ESPs and infected intestinal contents, wherein four were proven to show anti-inflammation properties, namely, L-glutamine, glutamine (Gln), pyruvate, and alanine-Gln (Ala-Gln). In summary, we have provided a method for the identification and analysis of parasite-derived molecules with potential anti-inflammatory properties in the present study. This array of anti-inflammatory metabolites could provide clues for future evaluation and translational study of these anti-inflammatory molecules.

## Introduction

As a soil-transmitted helminth, hookworm has been implicated in the incidence of several conditions, namely, iron deficiency anemia (IDA), malnutrition, and other chronic health problems, which are defined by intensity of infection in human host, wherein they can cause impaired physical and cognitive development as well as adverse outcome of pregnancy and lethargy (Loukas et al., 2016). Humans could be infected by three principal species of hookworm, viz., *Ancylostoma ceylanicum*, *Ancylostoma duodenale*, and *Necator americanus*, which complete their life cycle through skin penetration, pulmonary migration, and small intestine maturity in their host (Brooker et al., 2004). Hookworm infection remains an important health problem in areas with inadequate sanitation (namely rural subtropical and tropical countries), wherein it affects almost 500 million people with approximately 4.1 million annual loss of disability adjusted life years (DALYs) (Bartsch et al., 2016). Meanwhile, with a major burden of hookworm infection in areas described above, epidemiological evidence showed that a negative correlation was observed between hookworm infection and occurrence/frequency of inflammatory diseases such as metabolic disorders, allergic conditions, and inflammatory bowel disease (IBD), which could be defined as “hygiene hypothesis” (Maizels et al., 2004; Briggs et al., 2016; Ryan et al., 2020). Guided by this hypothesis, numerous studies of helminth-based therapy for different inflammatory disease models have been explored in recent years, wherein it was found that derived products of helminths showed drug-like anti-inflammatory activities in human, mice, and other larger animals (Summers et al., 2005; McSorley et al., 2012; Scholmerich et al., 2017). However, different mechanisms were elucidated in various models intervened by different species of helminth or derived molecules, including alternatively activated macrophages, mucus production, and wound repairing as well as regulatory cell population-induced abundance production of IL-10 and powerful drivers of type II immune responses (Harris and Loke, 2018; Lothstein and Gause, 2021). Moreover, microbiome and its metabolite interactions with helminths might also become increasingly important for anti-inflammatory mechanisms (Ramanan et al., 2016; Brosschot and Reynolds, 2018). Thus, identification and evaluation of helminth-derived molecules as anti-inflammatory agents are urgently needed for future translational research.

Considering the ethical concerns of live helminth infection, numerous helminth-derived molecules including proteins, lipids, and enzymes have been identified to potentially exert several functions of immunoregulation in different models of inflammatory disease (Lothstein and Gause, 2021). Ac-AIP-2 and Nb-DNase II, derived from two hookworm experimental models, namely, *Ancylostoma caninum* (hookworm in dog) and *N. brasiliensis* (rodent hookworm), could modulate innate immune responses and regulate dendritic cell development and Treg activation *in vitro* and *in vivo* (Navarro et al., 2016; Bouchery et al., 2020). As hookworm parasitizes in the small intestine of the host, hookworm-derived excretory and secretory products (ESPs) including soluble proteins, small molecules, and extracellular vesicles could collectively play an important role in host–pathogen interactions. However, previous studies mainly focused on hookworm-derived proteins or enzymes with immune regulation or anti-inflammatory properties. Only two papers focused on the non-protein small metabolites derived from *N. brasiliensis*, which identified an array of small metabolites with potential anti-inflammatory activities *in vitro* (Wangchuk et al., 2019; Yeshi et al., 2020). However, it is still unclear what might happen to the metabolic process *in vivo* during *N. brasiliensis* infection and/or whether these small molecules could also exist or act within the small intestinal environment of the host.

**Figure 1:**
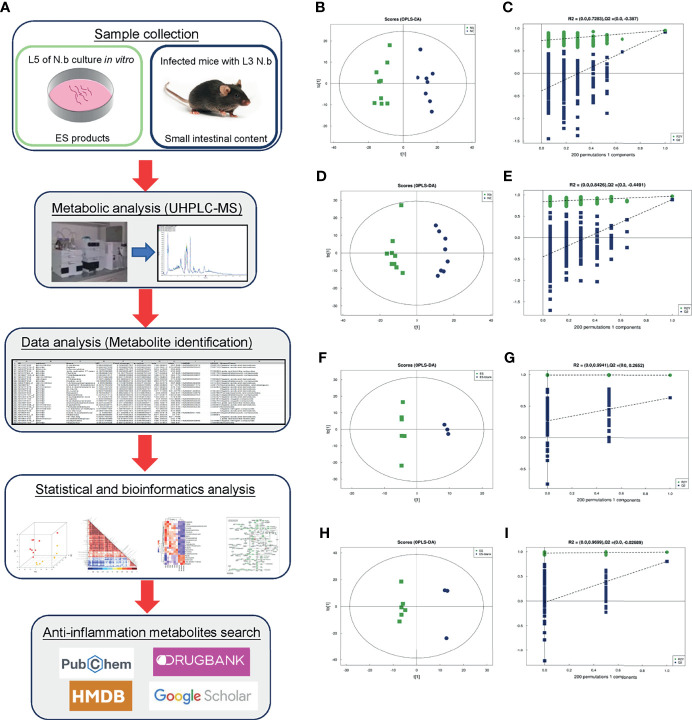
Schematic flowchart, OPLS-DA score plots, and corresponding permutation tests in positive and negative modes of the present study. Schematic flowchart of metabolomic analysis for ESPs and intestinal content from Nb-infected mice **(A)**. The scatter plots of the OPLS-DA score of ion mode: **(B, F)** positive and **(D, H)** negative. The results of the permutation test of ion mode: **(C, G)** positive and **(E, I)** negative.

Metabolomics, as one of the “omics” technologies, has egressed to become a powerful approach to comprehensively characterize metabolic alterations in recent times, wherein among other omics strategies, it is generally recognized as being closer and more representative of the phenotype (Nicholson et al., 2012; Guijas et al., 2018). Mass spectrometry (MS)-based analysis platform makes it possible to identify and quantify small-molecule metabolites from different tissue or body fluid samples, which possibly reflect the health or disease status and provide clues for disease diagnosis and treatment (Shah et al., 2015). For parasite infection, metabolomics analysis has been mostly applied for the discovery of biomarkers and the identification of a new drug target or intervention strategies (Legido-Quigley, 2010; Li et al., 2016; Huang et al., 2020). However, only very few studies focused on the identification of anti-inflammatory molecules during parasite infection. In the present study, we utilized a more sensitive metabolomics platform, ultra-high-performance liquid chromatography coupled with mass spectrometry (UHPLC-MS), to perform analysis of ESPs that were collected from cultured *N. brasiliensis* adult worm (*in vitro*) and small intestinal contents from *N. brasiliensis*-infected mice (*in vivo*), accordingly. Furthermore, we carried out a comparative analysis to explore common metabolites and reveal the *N. brasiliensis*-derived anti-inflammatory metabolites (as shown in **Figure 1A**).

## Materials and Methods

### Parasite, Animal, and Ethical Statement

*Nippostrongylus brasiliensis* (a kind gift by Professor Alex Loukas at James Cook University, Australia) was maintained and cycled in the laboratory of Jiangsu Institute of Parasitic Diseases (JIPD) according to a previous published reference (Camberis et al., 2003). C57BL/6 mice (5 weeks old, male) and Sprague–Dawley rats (about 300 g of weight each, male) that were provided by the Animal Center of JIPD (Wuxi, China) were used in the present study. Standard conditions (20–25°C and 12 h light–12 h dark) were maintained in the laboratory where the animals were housed amid unrestricted access to standard chow and water. The Ethical Committee for the Use of Experimental Animals at JIPD (Wuxi, China) gave approval to the protocol of the experiments.

### *Nippostrongylus brasiliensis* Culture and ESP Preparation

Sprague–Dawley rats were used to maintain *N. brasiliensis* and to collect adult worm as described previously (Camberis et al., 2003). Briefly, L3 stage larvae (3,500 larvae/rat) were used to infect the rats *via* the subcutaneous route, before they were euthanized with carbon dioxide asphyxiation on day 7 post-infection. Later, the rats were dissected to collect the adult worm from their small intestine. The small intestine was moved into a clean Petri dish containing 5 ml Dulbecco’s phosphate-buffered saline (DPBS) (Gibco-Thermo Fisher, MA, USA), prior to longitudinal slicing and cutting into smaller pieces. Afterwards, pieces of the intestines were placed on a gauze at the bottom of a funnel before it was filled with DPBS. Adult worms migrated out after 2 h of incubation (37°C) to settle at the underneath of the tube but retained debris of the intestinal contents of the host. The suspension containing worms was transferred from the bottom of the tube to a sterile centrifugated tube (50 ml), prior to washing twice with 20 ml of DPBS comprising antibiotic-antimycotic(5×) (Gibco-Thermo Fisher, MA, USA) amid counting under a dissecting microscope. Using 24-well (500 worms/well) plates, the culturing of adult worms was done for 7 days in RPMI-1640 medium (Gibco-Thermo Fisher, MA, USA) containing antibiotic-antimycotic(1×) under 37°C and 5% CO_2_ conditions. The cultured supernatant was collected every 24 h and replaced with fresh medium. Through centrifugation (at 2,000*g* and 4°C) for 10 min, the eggs and parasite fragments were removed and filtered with a 0.22-μm filter to obtain the final ESPs before storage at −80°C till it was used in a later experiment.

### Animal Infection and Sample Collection *In Vivo*

The C57BL/6 mice were used for infection and metabolomics analysis *in vivo*. Briefly, allocation of the mice into two groups (10 mice per group), namely, *N. brasiliensis* (Nb) and negative control (NC) groups, was done prior to the respective subcutaneous inoculation with L3 larvae of *N. brasiliensis* (500 larvae/mouse) or sterile saline (both in a 100-μl volume). On day 12 post-inoculation, the entire group of mice was euthanized and their small intestines were taken out under sterile conditions. The small intestinal content from each mouse was scraped into a 1.5-ml sterile tube, weighed, and preserved by storing at −80°C until further experiment.

### Analysis of Samples Using the LC-MS/MS Technique

An UHPLC (Agilent Technol-1290 infinity LC) that has been coupled to a time-of-flight (TOF) quadrupole platform (AB Sciex Triple-TOF 6600, Applied Protein Technol. Co. Ltd., Shanghai, China) was applied for further metabolomical analysis of the samples that were collected from ESPs and small intestinal contents of *N. brasiliensis*-infected mice coupled with negative controls, respectively (ES vs. ES-blank, Nb vs. NC). Also, separation of the samples with the HILIC technique was performed on a specialized column (ACQUIY-UPLC-BEH, Waters, Ireland). Mobile phase A consisted of ammonium acetate (25 mM)/aqueous ammonium hydroxide (25 mM), while mobile phase B consisted of acetonitrile in the positive and negative ESI modes. The mobile phase composition was altered as follows: for 1 min, B was maintained at 85% before linear reduction to 65% (within 11 min) and further reduction to 40% (within 0.1 minute) prior to keeping at this composition for 4 min. Later, the mobile gradient increased to 85% within 0.1 min but allowed a re-equilibration period of 5 min. Separation of the samples *via* the RPLC system was carried out on the Waters column (ACQUIY-UPLC-HSS T3, Ireland). The composition of the mobile phase for the positive ESI mode (positive) was water comprising formic acid (0.1%)—A and acetonitrile containing formic acid (0.1%)—B, while that of the negative ESI mode was made up of aqueous ammonium fluoride (0.5 mM)—A and acetonitrile—B. Gradient elution was performed by maintaining B at 0.1% for 1.5 min before linear increase to 99% within 11.5 min prior to further keeping the gradient for 3.5 min. Afterwards, the gradient was reduced to 1% within 0.1 min and allowed a re-equilibration period of 3.4 min. The temperature of the column was maintained at 25°C, while 0.3 ml/min was the flow rate of the mobile phases with sample injected in aliquots (2 µl). The following are the conditions of the ESI source: curtain gas (CUR), 30; gas 1, 60; gas 2, 60; source temperature, 600°C; and ion-spray voltage floating (ISVF), ± 5,500 V. The mass/charge (*m*/*z*) range for MS (for acquisition only) was set over 60–1,000 Da, while 0.20 s/spectra was the TOF-MS scan accumulation time. Likewise, the *m*/*z* range for the MS/MS auto method for acquisition was set over 25–1,000 Da with 0.05 s/spectra being the product ion scan (PIS) accumulation time. Acquisition of PIS was carried out in high sensitive mode through information-dependent acquisition (IDA). The following parameters were fixed accordingly: 35 V with ±15 eV as collision energy (CE) and −60 V (−) and 60 V (+) as declustering potential (DP), with the exclusion of 4 Da isotopes and monitoring of ion candidate per cycle (set at 10).

### Processing of Data

Prior to importation to XCMS software (freely available), the conversion of the raw data of MS (from.wiff file to.mzXML file) was carried out with Proteo-Wizard MSConvert. Parameters such as 25 ppm for cent-Wave *m*/*z*, c(10, 60) for peak-width, and c(10, 100) for pre-filter were used for picking the peaks. Likewise, grouping of the peaks was done using the accompanying parameters, viz., mzwid (0.025), minfrac (0.5), and bw (5). Isotopic and adduct annotations were performed with the collection of algorithms of metabolite profile annotation (CAMERA). Technically, variables that had more than 50% of the non-zero measurement values (in at least one group) were kept and employed for the extracted ion features. We established a database in our lab using reliable standards there were readily available to identify metabolites *via* comparison with accurate *m*/*z* value (less than 25 ppm) and spectra of MS/MS.

### Statistical Analysis

The R package (Ropls) was used to analyze the processed data after sum normalization. Multivariable analysis of data was performed, which was comprised of orthogonal partial least squares discriminant analysis (OPLS-DA) and principal component analysis (PCA) coupled with Pareto scaling. Evaluation of model robustness was done with the seven-fold cross-validation and response permutation testing. Using the OPLS-DA model, the variable importance in projection (VIP) score of each variable was computed to show the contribution of VIP to the classification. Determination of significant differences within two groups of independent measurements was statistically carried out with Student’s *t-*test. Significantly changed metabolites were screened when *p <*0.05 and VIP >1. Correlation between two variables was analyzed using Pearson’s correlation analysis.

Differentially expressed metabolites were analyzed through the metabolomics pathway using MetaboAnalyst. Presentation of Nb infection-associated pathways was done based on pathway impact and *p-*values derived from pathway topology and pathway enrichment analyses, respectively.

### Comprehensive Literature Searches and Content Analyses of Metabolites With Pharmacological Effects

Literature searches and database survey were comprehensively conducted (for each metabolite) on the account of the list of differential metabolites that were identified from the Nb and ES groups. Databases that were searched included the Human Metabolome Database (HMDB, comprising metabolite entries of 114,100, including metabolites that are soluble in lipid and water) (Wishart et al., 2013) and PubChem (Kim et al., 2016). Additionally, references that were relevant to the biological properties of each metabolite were identified through Google Scholar. Anti-inflammatory, biological activities, and immune regulation were the specific keywords that ensured a unique search. Performance of database and reference content analyses focused on previously reported biological properties, which was subsequently tabularized and quoted against each substance.

## Results

### System Stability Assessment

In this experiment, changes in the metabolic profile of the samples were analyzed through metabolomics methods on the account of UHPLC-Q TOF-MS technology. As shown by the chromatographic total ion (TIC) technique of QC samples, overlapping of the intensity and retention time of each chromatographical peak was observed. Analysis of peaks that were excerpted from the entire experimental and QC samples was performed using the PCA method with results shown in **Supplementary Figure S1**, wherein outliers were obviously seen in the samples of positive and negative ion modes, while those of the QC were clustered closely. The results suggest the stability of the system of instrumental analysis used in this experiment and the reliability of the experimental data obtained. Thus, the observed metabolic spectrum differences during the experiment could reflect the biologic differences between the samples.

### Multivariate Statistical Analysis of Metabolite Profiling

After multivariate pattern recognition analysis of OPLS-DA, the metabolic profile of Nb was observed to be distinct from that of NC. Thus, the OPLS-DA model (**Figure 1**) could clearly distinguish between Nb and NC. The heatmap constructed from 17 samples of mice (**Figures 3A, B**) supported the aforementioned observation. Testing of the robustness of the model showed a good predictive model performance (for the negative ion mode: *R* ^2^ *Y* = 0.842 and *Q* ^2^ = 0.608, for the positive ion mode: *R* ^2^ *Y* = 0.986 and *Q* ^2^ = 0.956) but was not overfitting (**Figures 1C, E**), indicating that Nb infection may induce obvious alterations in the metabolism of the mice.

Similarly, a supervised analysis of the ES group was done with OPLS-DA analysis. Observation of the OPLS-DA score plots showed a clear discrimination between the ES and the control groups. The permutation tests verified the validity of the model (for the positive ion mode: *R* ^2^ *Y* = 0.996 and *Q* ^2^ = 0.637, for negative ion mode: *R* ^2^ *Y* = 0.995 and *Q* ^2^ = 0.807) (**Figures 1G, I**). Subsequently, heatmaps of ES metabolic characteristics were carried out to view the data more intuitively (**Figures 3C, D**). The results of heatmaps were consistent with those of OPLS-DA. Overall, the above results indicate that the adult worms secreted several small molecules.

### Screening and Analysis of Differential Metabolites

**Figure 2:**
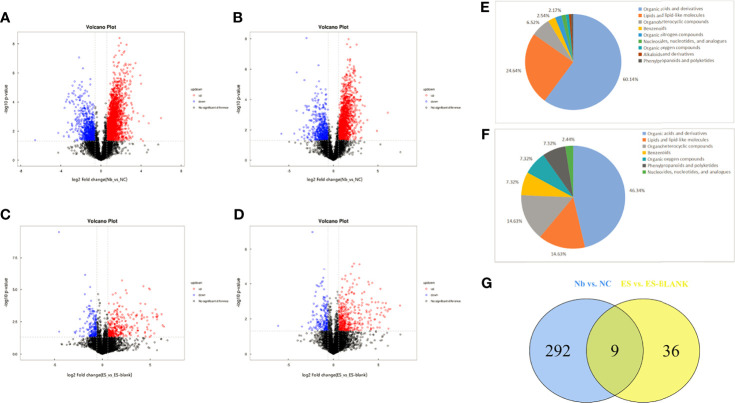
Unique and common differential metabolites in Nb vs. NC and ES vs. ES-blank groups. The volcano maps of differential metabolites of positive **(A, C)** and negative **(B, D)** ion modes. Red scatters denote the upward trend in metabolites, blue scatters indicate the downward trend in metabolites, and black scatters show the non-significant trend in metabolites. The chemical classification chart of distinct metabolites in the Nb **(E)** and ES groups **(F)** as well as common and unique metabolite distribution within the two sample groups **(G)**. Different colors in the picture indicate distinct chemical classifications. Percentage represents the proportion of the metabolite number in the chemical classification to the total metabolite number. The Venn diagram shows the overlap of the significantly different metabolites. Two circles were used to denote the clustering of the entire distinctly expressed metabolites into two comparison groups. The number of distinctly expressed metabolites in one comparison group was taken as the sum of the entire figures represented in one circle.

Through fold change (FC) and *t*-test methods, the volcano plot analysis was used to identify distinctive metabolites. Easy isolation of Nb infection-induced metabolites and identification of excretory secretions of adult worms were carried out with the help of the volcano plots. As shown in **Figure 2**, points that have the greatest degrees of difference are those found at the two extremes of the plot. Log_2_(FC > 1.5 or <0.67) was plotted on the *x*-axis with −log_10_(*p*-value, derived from *t*-test) on the *y*-axis (**Figure 2**).

Calculation of VIP score for the individual variables in the OPLS-DA model was done to assess the VIP contribution to classification. Obviously, a total of 301 metabolites exhibited abundance shifts after the mice were infected with Nb, including downregulated (71) and upregulated (230) metabolites. In totality, 45 different metabolites were screened among the ES/ES-blank, wherein 16 were downregulated, while 29 were upregulated. **Supplementary Table S1** shows detailed information of individual metabolites such as fold change analysis, HMDB-ID, and VIP values.

Different metabolites in the Nb group belong to nine different chemical classification categories/groups with seven in the ES group (**Figures 2E, F**). Three categories that had the highest differential metabolite content among the Nb and ES groups were mainly organic acids and their derivatives, followed by lipid-like and lipid compounds and organoheterocyclic molecules.

**Figure 3:**
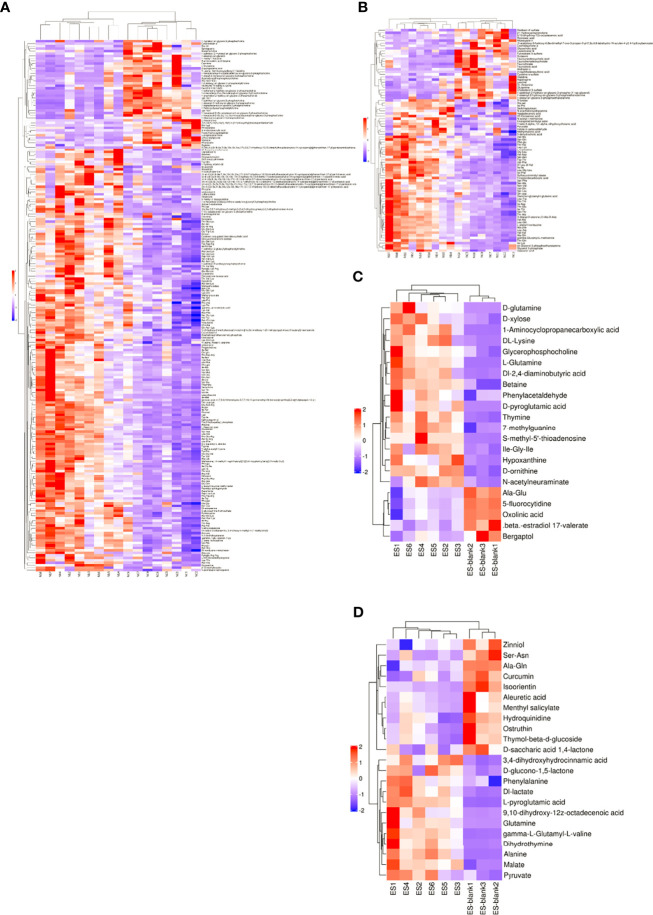
Heatmaps of different metabolites within the Nb and ES groups. **(A, C)** show the positive ion mode and **(B, D)** show the negative ion mode. The significance of metabolite change (red denotes upregulated and blue indicates downregulated) was proportional to the color of each section. Rows correspond to metabolites, while columns correspond to samples.

In addition, the difference in the expression of patterns of metabolites in various samples was assessed with hierarchical cluster analysis heatmap (**Figure 3**). Importantly, clustering heatmaps can more intuitively show the relationship between samples. Usually, the variation patterns of metabolome compositions are clearly shown by the heatmap, wherein they are obviously separated from the control. Exemplarily, alanine glutamine and pyruvate were abundantly present in the ESPs of Nb but absent in the culture medium.

Nine metabolites were found to be common with regard to their differential features when differences in their metabolic profiles were compared, namely, Nb vs. NC and ES vs. ES-blank (**Figure 2G**), wherein these metabolites comprised mostly products of organic acids and derivatives, followed by organoheterocyclic molecules as well as lipid-like and lipid compounds (**Table 1**).

**Table 1:** List of the common differential metabolites in Nb vs. NC and ES vs. ES-blank.

ESI mode	Metabolites	Superclass	*m*/*z*	rt (s)	ES vs. ES-blank		Nb vs. NC	
					VIP	*p*-value	VIP	*p*-value
+	L-Glutamine	Organic acids and derivatives	169.057	373.919	7.297	0.002	1.028	0.006
+	Ile-Gly-Ile	Organic acids and derivatives	302.205	211.549	1.703	0.009	2.989	0.000
+	Thymine	Organoheterocyclic compounds	127.050	97.825	2.059	0.001	7.988	0.006
+	D-pyroglutamic acid	Organic acids and derivatives	130.049	374.519	10.833	0.015	2.103	0.018
−	Glutamine	Organic acids and derivatives	145.062	374.131	7.870	0.036	2.012	0.009
−	Pyruvate	Organic acids and derivatives	87.008	130.075	1.791	0.049	1.237	0.001
−	Ala-Gln	Organic acids and derivatives	216.099	354.041	10.097	0.002	1.357	0.001
−	Ser-Asn	Organic acids and derivatives	218.078	368.522	1.282	0.006	1.013	0.021
−	9,10-Dihydroxy-12z-octadecenoic acid	Lipids and lipid-like molecules	313.239	78.357	3.041	0.031	5.962	0.015

ESI mode: +, positive ion mode; −, negative ion mode.

m/z, mass-to-charge ratio; rt, retention time; VIP, variable importance in the projection; FC, fold change.

Comprehensive literature search coupled with database survey discovered 20 small molecules with anti-inflammatory activity with **Table 2** showing the retention times, *m*/*z*, and chemotaxonomy, alongside the described biological activities of the metabolites. Besides anti-inflammatory activity shown in **Table 2**, the associated activities of metabolites were also discovered including improved intestinal immunity (Ala-Gln, phenylalanine) and neuroprotective (betaine, curcumin), antitumor (betaine, curcumin), antidiabetic (betaine), antiproliferative (curcumin), anti-aging (curcumin), antioxidant (irbesartan), and gastroprotective properties (gamma-aminobutyric acid).

**Table 2:** Summary table of differential metabolites with pharmacological activity.

Name	Superclass	*m*/*z*	rt (s)	Reported pharmacological activities	ES		Nb	
					VIP	*p*-value	VIP	*p*-value
Ala-Gln	Organic acids and derivatives	216.099	354.041	Improved intestinal immunity (Shimizu and Son, 2007; Araújo et al., 2015); anti-inflammatory (Cruzat et al., 2015; Foschetti et al., 2020; Liu et al., 2020)	10.097	0.002	1.357	0.001
Pyruvate	Organic acids and derivatives	87.008	130.075	Anti-inflammatory (Wang et al., 2009; Yang et al., 2016)	1.791	0.049	1.237	0.001
Glutamine	Organic acids and derivatives	145.062	374.131	Anti-inflammatory (Ren et al., 2013)	7.87	0.036	2.012	0.009
L-Glutamine	Organic acids and derivatives	Su	373.919	Anti-inflammatory (Almeida et al., 2020; Paixao et al., 2021)	7.297	0.002	1.028	0.006
Betaine	Organic acids and derivatives	118.085	274.605	Neuroprotective (Singhal et al., 2020); antitumor (Kim et al., 2014); antidiabetic (Jiang et al., 2019); anti-inflammatory (Yang et al., 2018)	5.896	0.000		
Bergaptol	Phenylpropanoids and polyketides	203.051	302.917	Anti-inflammatory (Shen et al., 2020)	3.794	0.014		
Gamma-L-glutamyl-L-valine	Organic acids and derivatives	227.104	377.969	Anti-inflammatory (Chee et al., 2017; Guha et al., 2020)	1.659	0.002		
Curcumin	Phenylpropanoids and polyketides	367.106	305.841	Antiproliferative (Chainoglou and Hadjipavlou-Litina, 2019); anti-aging (Zia et al., 2021); antitumor (Carroll et al., 2011; Feng et al., 2017); neuroprotective (Vecchi Brumatti et al., 2014; Yu et al., 2018); anti-inflammatory (Ammar el et al., 2011; Ma et al., 2017)	15.442	0.011		
Phenylalanine	Organic acids and derivatives	164.072	257.387	Improved intestinal immunity (Feng et al., 2015); anti-inflammatory (Neurauter et al., 2008)	1.902	0.026		
D-saccharic acid 1,4-lactone	Organoheterocyclic compounds	190.995	307.027	Anti-inflammatory (Bhattacharya et al., 2013)	1.013	0.043		
Olanzapine		157.096	252.236	Anti-inflammatory (Sugino et al., 2009; Faour-Nmarne and Azab, 2016; Stapel et al., 2018)			1.452	0.001
Papaverine	Organoheterocyclic compounds	340.145	370.151	Anti-inflammatory (Alves de Almeida et al., 2017; Leem et al., 2021)			1.083	0.001
Carnitine	Organic nitrogen compounds	162.111	356.015	Anti-inflammatory (Chittur et al., 2011; Orsal et al., 2013)			5.629	0.001
Thiamine	Organoheterocyclic compounds	283.124	360.263	Anti-inflammatory (Pan et al., 2017; Belsky et al., 2018; Marik, 2018; Ma et al., 2021)			2.017	0.002
Pregnenolone	Lipids and lipid-like molecules	317.245	32.036	Anti-inflammatory (Vallée et al., 2014; Vallée, 2016; Murugan et al., 2019)			1.21	0.006
Adenine	Organoheterocyclic compounds	136.06	142.232	Anti-inflammatory (Fukuda et al., 2017; Silwal et al., 2018)			2.228	0.007
Irbesartan	Benzenoids	429.258	151.72	Antioxidant (El-Said et al., 2019); anti-inflammatory (Yisireyili et al., 2018; Zhong et al., 2020; Nijiati et al., 2021)			2.962	0.008
PC(16:0/16:0)	Lipids and lipid-like molecules	756.548	147.302	Anti-inflammatory (Treede et al., 2007; Treede et al., 2009; Chen et al., 2019)			2.02	0.022
Gamma-aminobutyric acid	Organic acids and derivatives	180.1	38.032	Gastroprotective (Xie et al., 2017); anti-inflammatory (Choi et al., 2012; Huang et al., 2019; Ngo and Vo, 2019)			2.328	0.046
Arginine	Organic acids and derivatives	175.117	301.295	Anti-inflammatory (Holen et al., 2014; Badurdeen et al., 2015; Yue et al., 2015; Birmani et al., 2019)			1.583	0.048

### Analyses of the Biosynthetic and Metabolic Pathways of the Identified Compounds

**Figure 4:**
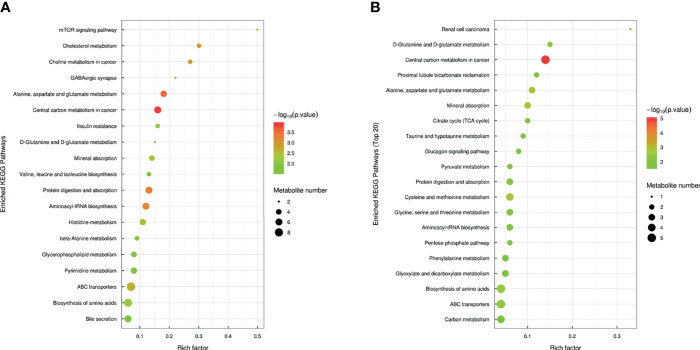
Bubble plot of the pathway analysis of Nb vs. NC **(A)** and ES vs. ES-blank **(B)**. A metabolic pathway is represented by each bubble in the bubble chart. In the topological analysis, the pathway is influenced by factors such as the size and abscissa of the bubble. The ordinate and color of the bubble with the latter indicating the *p*-value of the enrichment analysis. The darker the color, the smaller the *p*-value and the more significant the enrichment degree. The rich factor represents the ratio of the different metabolites in the pathway to the detected metabolites.

Through KEGG pathway analysis for the related biological effects and pathways of distinctly expressed metabolites, the determined metabolic pathway enrichment diagram (**Figure 4**) suggests that eight similar metabolic pathways were observed in the Nb and ES groups. These include metabolism of central carbons in tumor; metabolism of glutamate, aspartate, and alanine; digestion and absorption of proteins; absorption of minerals; biosynthesis of amino acids; metabolism of glutamine; and biosynthesis of amino-acyl tRNA and transporters of ATP binding cassette. **Figure 4** displays the perturbed metabolic pathways in samples of intestinal content, thereby depicting bile secretion; mTOR signaling pathway; insulin resistance; GABAergic synapse; biosynthesis of isoleucine, leucine, and valine; and metabolism of beta-alanine, cholesterol, pyrimidine, histidine, and glycerophospholipids, which were significantly enriched in the infected mice compared with the healthy control. Meanwhile, metabolism of pyruvate, glutathione, cysteine, methionine, threonine, glycine, and serine as well as bicarbonate reclamation in the proximal tubules; glucagon signaling pathway; type II diabetes mellitus; citrate cycle (TCA cycle); and other metabolic pathways were significantly affected in the ES group.

## Discussion

Available evidence suggests that hookworm could exert an anti-inflammation property for their long-term survival in human or animal host, which may in turn provide a beneficial effect against inflammatory-related diseases (Ferreira et al., 2017; Lothstein and Gause, 2021). Consequently, screening and identification of hookworm-derived molecules with pharmacological activity will be highly investigated in the near future. Besides genomic and proteomic techniques, the application of the metabolomic platform to the pharmaceutical field has begun in recent years, wherein it could screen and identify non-protein small metabolites, thereby reflecting metabolic changes in the host (Wangchuk et al., 2019; Yeshi et al., 2020). The difficulty in obtaining and maintaining human hookworms in the laboratory is a major challenge in human hookworm studies. In view of the similarity of life cycle and pathogenicity to human hookworm, *N. brasiliensis* has been defined as a model and is widely used in human hookworm research especially in immunobiology, drug screening, etc. (Dominguez et al., 2000; Camberis et al., 2003). Therefore, *N. brasiliensis* was employed as a model to carry out two independent metabolomics analyses in order to explore anti-inflammatory molecules that were derived from *N. brasiliensis* through *in-vitro* and *in-vivo* pathways in the current study.

Principal molecules of helminths constitute the ESPs and parasite external surface (Hewitson et al., 2009). As a key boundary between the hosts and helminthic parasites, ESPs are usually secreted as a mixture of carbohydrates, lipids, and proteins from the outer surface or oral orifice of the parasite (Pearson et al., 2012). We analyzed ESPs of *N. brasiliensis* in our study, wherein 10 out of 45 differential metabolites were discovered to demonstrate potential anti-inflammatory properties as described in published works. These metabolites could be subclassed into amino acids, furanocoumarins, linear diarylheptanoids, gamma butyrolactones, and alpha-keto acids. Although glutamine and phenylalanine have also been detected in previous research through GC-MS-based platform (Wangchuk et al., 2019), the other eight metabolites have not been found in other previous published works, which may be ascribable to the application of different metabolomical platforms. Furthermore, through non-targeted liquid chromatography mass spectrometry (LC-MS) platform, metabolites such as betaine and L-glutamine were also found to be in ESPs of *N. brasiliensis* L3 stage (Yeshi et al., 2020). Therefore, standardization of metabolomics analysis for ESPs of hookworm should be established in the future to increase the credibility of the results.

In addition to *in-vitro* work on the ESPs of cultured adult worm, the *in-vivo* study on the *N. brasiliensis*-parasitized mouse small intestines could provide direct information on host–parasite interaction (Wangchuk et al., 2019). In the present study, we carried out metabolomics analysis of the intestinal contents of *N. brasiliensis*-infected mouse for the first time, and our results revealed that 301 metabolites were differentially expressed. Through literature searching, we discovered 14 metabolites with anti-inflammatory or antioxidative properties which could be subclassed into amino acids, benzylisoquinolines, quaternary ammonium salts, pyrimidines, pregnane steroids, purines, biphenyls, and glycerophosphocholines. For example, as a candidate for anti-inflammation, glutamine could crucially influence the long-term treatment outcome of inflammatory conditions by regulating inflammation through pathways such as mitogen-activated protein-kinases (MAPK), signal transducer and activator of transcription (STAT), and nuclear factor-kappa B (NF-κB) (Ren et al., 2013). Also, existing evidence has shown that in response to vaccination against the influenza virus, L-glutamine could improve the immunity of the mucosa by modulating the salivary cytokine profile (IL-6 and IL-10) and increasing SIgA levels in the upper airways (Paixao et al., 2021). Irbesartan could ameliorate inflammation and fibrosis in the hypertensive renal injury model through inhibiting Th22 cell chemotaxis and infiltration as well as CCL20, CCL22, and CCL27 expression (Zhong et al., 2020). Papaverine could inhibit the activation of NLRP3 inflammasome by modulating NF-kappa B and CREB signaling pathways, which results in reduced microglial activation and neuronal cell death (Leem et al., 2021). Olanzapine treatment could decrease in expression and secretion of IL-1beta and TNF-alpha significantly in *ex vivo* stimulation of primary human peripheral blood mononuclear cells (Stapel et al., 2018). All these suggest that a switch to anti-inflammation status of host immune system might have occurred with the production of numerous anti-inflammatory metabolites during *N. brasiliensis* infection in mice. This could further confirm the inhibitory role of inflammatory responses following infection by the parasites.

To further explore the source of the differential metabolites, especially those with anti-inflammatory properties, we compared the differential metabolites from ESPs and intestinal contents of mice that were infected by *N. brasiliensis*. We found that nine metabolites co-existed in two different samples which we speculated to originate from *N. brasiliensis*. Furthermore, four metabolites of the discovered nine common metabolites had anti-inflammatory properties, and they included L-glutamine, glutamine, pyruvate, and Ala-Gln. For instance, pyruvate, a key intermediate in glucose metabolism, could exert an anti-inflammation role in models of experimental stroke and inflammation, as well as systemic inflammation and multiple dysfunctions (Wang et al., 2009; Yang et al., 2016). Ala-Gln, short for the dipeptide alanyl-glutamine, could attenuate inflammation in various experiments including intestinal mucositis, *Escherichia coli* lipopolysaccharide-induced vascular hyporeactivity, asthma, obesity-associated diabetes, and concomitant inflammatory *via* sirtuin 1/HUR signaling in β cells (Jing et al., 2007; Araújo et al., 2015; Cruzat et al., 2015; Liu et al., 2020). Besides the *N. brasiliensis*-derived anti-inflammatory metabolites, the host-derived metabolites might be more important for anti-inflammation roles after *N. brasiliensis* infection as demonstrated by the other 10 anti-inflammatory metabolites that were also discovered in this work. However, the other metabolites detected in the present study might also have anti-inflammatory effects or other pharmacological activities which require further research. On the other hand, the trilateral relationship among parasite, microbiota, and host cells should also be taken into consideration (Midha et al., 2021), which indicates that intestinal microbiota may also play a crucial role for the alteration of metabolites during *N. brasiliensis* infection in a mouse model, albeit a comprehensive investigation is needed for a clear understanding of this interaction.

In summary, we have established a method for the analysis of parasite-derived anti-inflammatory molecules and revealed an array of metabolites with anti-inflammatory activities through the UHPLC-MS platform, which could therefore provide clues for the further identification, evaluation, and translation of such metabolites in the not too distant future. However, *N. brasiliensis*, a model of human hookworm, is different from human hookworm in terms of species taxonomy and parasitism as reported above (Camberis et al., 2003). How to translate *N. brasiliensis*-based study results to the pharmacological properties of human hookworm will be another question that needs to be interrogated in the future.

## Data Availability Statement

The original contributions presented in the study are included in the article and supplementary material. Metabolomics data have been deposited to the EMBL-EBI MetaboLights database (doi: 10.1093/nar/gkz1019, PMID:31691833) with the identifier MTBLS3486. The complete dataset can be accessed here https://www.ebi.ac.uk/metabolights/MTBLS3486.

## Ethics Statement

The animal study was reviewed and approved by the Ethical Committee for the Use of Experimental Animals at Jiangsu Institute of Parasitic Diseases (Approval Number: JIPD-2020-007).

## Author Contributions

YD and JW conceived and designed the study. YYC, XD, MZ, YY, and YJC performed the experiments and collected the data. YYC, QZ, MZ, and YF analyzed the data. YD, YYC, and JW wrote and revised the manuscript. All authors contributed to the article and approved the submitted version.

## Funding

This work was supported by the National Key R&D Program of China (2020YFC1200100), the Jiangsu Provincial Department of Science and Technology (BM2018020), the National Natural Science Foundation of China (NSFC 82170269), the Jiangsu Province’s Key Provincial Talents Program (ZDRCB2016005), the Foundation of Clinical Medical Research Center of Yili Autonomous Prefecture (YL2020ms09), and Jiangsu Provincial Project of Invigorating Health Care through Science, Technology and Education and the Jiangsu Provincial Commission of Health.

## Conflict of Interest

The authors declare that the research was conducted in the absence of any commercial or financial relationships that could be construed as a potential conflict of interest.

## Publisher’s Note

All claims expressed in this article are solely those of the authors and do not necessarily represent those of their affiliated organizations, or those of the publisher, the editors and the reviewers. Any product that may be evaluated in this article, or claim that may be made by its manufacturer, is not guaranteed or endorsed by the publisher.

## Supplementary Material

Supplementary Figure 1 Evaluation of the quality of the experiment. The total ion chromatography (TIC) of the QC sample in modes of positive ion **(A)** and negative ion **(B)**. Retention time is denoted by *x*-axis bars, while total ionic strength is represented by *y*-axis bars. PCA score plots of all experimental samples in the modes of positive ion **(C)** and negative ion **(D)**.
