# Supplementary figures and images for: Mining Anti-Inflammation Molecules From Nippostrongylus brasiliensis-Derived Products Through the Metabolomics Approach
Source: Front Cell Infect Microbiol. 2021 Nov 11;11:781132. doi: 10.3389/fcimb.2021.781132 (PMC8632049; doi:10.3389/fcimb.2021.781132)

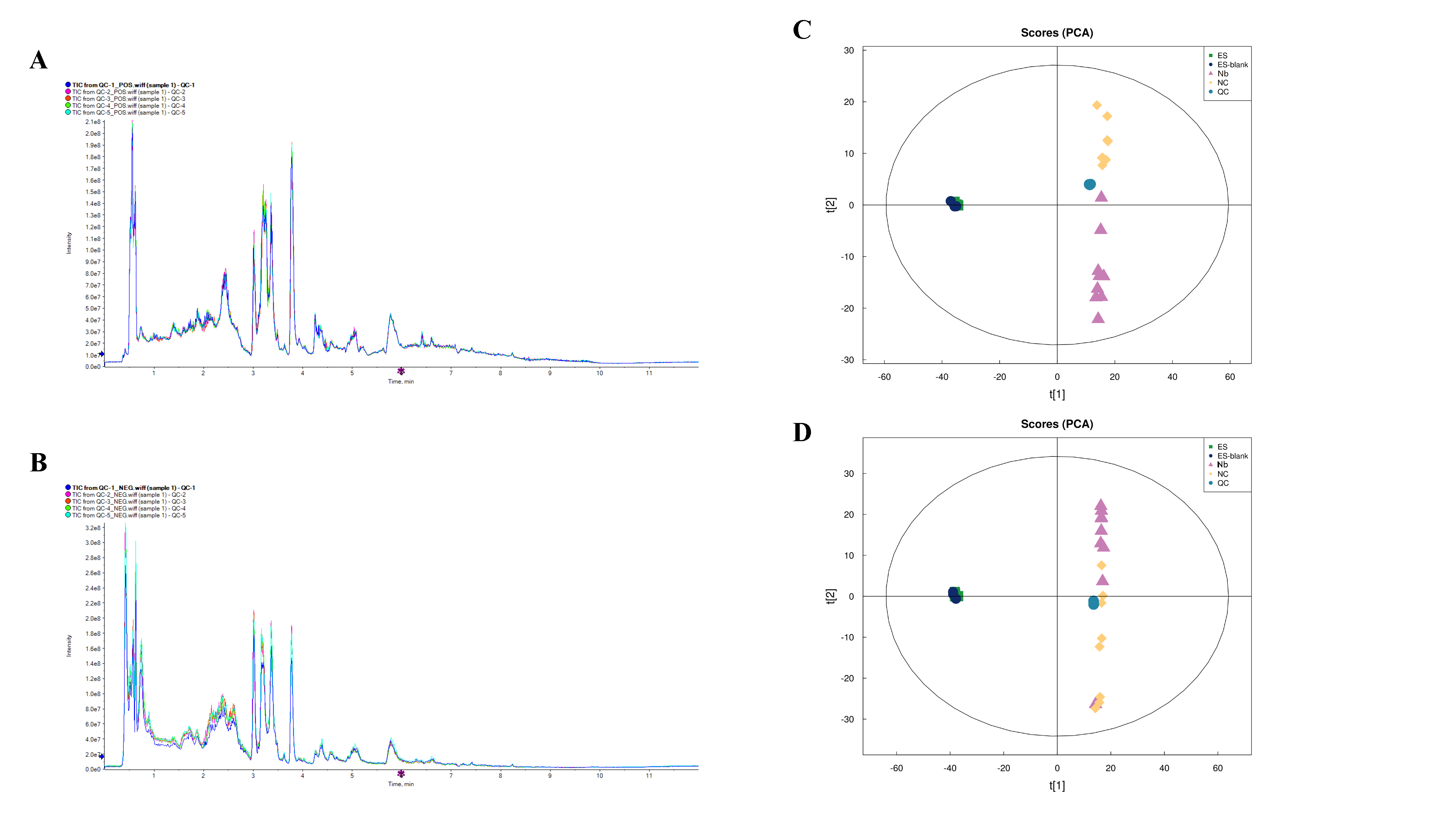

Supplement: Supplementary Figure 1 — Evaluation of the quality of the experiment. The total ion chromatography (TIC) of the QC sample in modes of positive ion (A) and negative ion (B). Retention time is denoted by x-axis bars, while total ionic strength is represented by y-axis bars. PCA score plots of all experimental samples in the modes of positive ion (C) and negative ion (D). [file Image_1.tiff]
